# Selective initial in vivo homing pattern of 5T2 multiple myeloma cells in the C57BL/KalwRij mouse

**DOI:** 10.1054/bjoc.1999.1024

**Published:** 2000-02

**Authors:** K Vanderkerken, C De Greef, K Asosingh, B Arteta, M De Veerman, I Vande Broek, I Van Riet, M Kobayashi, B Smedsrod, B Van Camp

**Affiliations:** Departments of Haematology and Immunology and Physiology, Free University Brussels, Laarbeeklaan 103, Brussels, B-1090, Belgium; Department of Experimental Pathology, University of Tromso, Tromso, N-9037, Norway; Laboratory of Pathology, Hokkaido University School of Medicine, Sapporo, 060-8638, Japan

**Keywords:** homing, multiple myeloma, bone marrow

## Abstract

One of the main characteristics of multiple myeloma cells is their predominant localization in the bone marrow. It is, however, unclear whether this is due to a selective initial entry, or whether this entry is more random and other processes like survival and/or growth stimulation, only present in the medullar microenvironment, are unique. To investigate this, in vivo homing kinetics of murine 5T2MM cells shortly after injection were assessed in bone marrow, liver, spleen, lungs, heart, intestines, kidney and testis by tracing of radiolabelled cells, by immunostaining of isolated cells and by polymerase chain reaction analysis. We demonstrated the presence of 5T2MM cells in bone marrow, spleen and liver with all other organs being negative. Adhesion assays of 5T2MM cells to different types of endothelial cells demonstrated a selective adhesion of 5T2MM cells to bone marrow and liver and not to lung endothelial cells. We here demonstrate that the specific in vivo localization of the 5T2MM cells is a result of the combination of a selective entry/adhesion of the 5T2MM cells in the bone marrow, spleen and liver, and a selective survival and growth of these tumour cells in the bone marrow and spleen but not in the liver. © 2000 Cancer Research Campaign


*British Journal of Cancer* (2000) **82**(4), 953–959
© 2000 Cancer Research Campaign
DOI: 10.1054/ bjoc.1999.1024, available online at http://www.idealibrary.com on IDEAL®

### Selective initial in vivo homing pattern of 5T2 multiple myeloma cells in the C57BL/KalwRij mouse

**K Vanderkerken[1], C De Greef[1], K Asosingh[1], B Arteta[3], M De Veerman[2], I Vande Broek[1], I Van Riet[1], M Kobayashi[4], B Smedsrod[3] and B Van Camp[1]**

Departments of [1]Haematology and Immunology, and [2]Physiology, Free University Brussels, Laarbeeklaan 103, B-1090 Brussels, Belgium; [3]Department of Experimental Pathology, University of Tromso, N-9037 Tromso, Norway; [4]Laboratory of Pathology, Hokkaido University School of Medicine, Sapporo 060-8638, Japan

**Summary** One of the main characteristics of multiple myeloma cells is their predominant localization in the bone marrow. It is, however, unclear whether this is due to a selective initial entry, or whether this entry is more random and other processes like survival and/or growth stimulation, only present in the medullar microenvironment, are unique. To investigate this, in vivo homing kinetics of murine 5T2MM cells shortly after injection were assessed in bone marrow, liver, spleen, lungs, heart, intestines, kidney and testis by tracing of radiolabelled cells, by immunostaining of isolated cells and by polymerase chain reaction analysis. We demonstrated the presence of 5T2MM cells in bone marrow, spleen and liver with all other organs being negative. Adhesion assays of 5T2MM cells to different types of endothelial cells demonstrated a selective adhesion of 5T2MM cells to bone marrow and liver and not to lung endothelial cells. We here demonstrate that the specific in vivo localization of the 5T2MM cells is a result of the combination of a selective entry/adhesion of the 5T2MM cells in the bone marrow, spleen and liver, and a selective survival and growth of these tumour cells in the bone marrow and spleen but not in the liver. © 2000 Cancer Research Campaign

**Keywords:** homing; multiple myeloma; bone marrow

Multiple myeloma (MM) is a B-cell neoplasm, mainly characterized by the monoclonal expansion of plasma cells, the presence of monoclonal serum immunoglobulin and the occurrence of osteolysis. The malignant cell corresponds to a long-lived plasma cell located in the bone marrow (BM) carrying somatically rearranged Ig genes with clonally fixed hypervariable regions (Bakkus et al, 1992; Hallek et al, 1998). This implies a post-germinal centre origin of the MM cells (Bakkus et al, 1992) having a specific migration to the extravascular compartment of the BM. This process of migration of MM cells to the extravascular compartment of the BM, and referred to as ‘homing’, is believed to be highly specific since no elevated quantity of malignant B-cells are observed in the peripheral blood or in other organs. After their specific homing to the BM, these cells proliferate and differentiate into mature plasma cells. It is only at the endstage of the disease of some patients that extramedullar spread is observed.

In our previous work we have reported the 5T2MM model in the C57BL/KaLwRij mice (Radl et al, 1979) as a good model to study the homing of human myeloma cells in the BM (Vanderkerken et al, 1996, 1997). In this model, MM cells are isolated from diseased animals and are transplanted into young syngeneic recipients by intravenous injection. From 9 weeks on after injection we observe a specific localization of the MM cells in the BM and partly in the spleen of the mice. This study did not, however, reveal whether this selective localization of MM cells in the BM is due to a selective initial entry of the cells through the endothelial barrier of the BM, or whether this homing is more random, and survival and growth factors, only present in BM, determine the unique presence of MM cells.

The migration of lymphocytes in general (Butcher and Picker, 1996) is known to include a multistep cascade of processes mainly involving the endothelium. This interaction with the endothelium requires at least four independent steps: initial tethering, arrest, adhesion and transendothelial migration. It is the combination of the selectivity of each independent step which makes the process highly specific. In vivo, BM endothelial cells (BM EC) act as gate-keepers separating the BM compartment from the sinusoidal lumen. These cells may therefore play an important role in the selective entry of the MM cells.

We conclude here that MM cells have a selective homing behaviour which is the result of the combination of a selective adhesion to the BM EC followed by the entry in the extravascular compartment and a selective survival and growth, making the BM and spleen microenvironment unique.

### MATERIALS AND METHODS

### Mice

C57BL/KaLwRijHsd mice (Harlan CPB Zeist, The Netherlands) were housed under conventional conditions and had free access to tap water and food. They were anaesthetized by intraperitoneal (i.p.) injection of 200 μl Imalgene (80 mg $kg^{-1}$ body weight; Rhône Merieux, Lyon, France) and Rompoun mixture (10 mg $kg^{-1}$ body weight; Bayer, Sint-Truiden, Belgium). Mice were killed by carbon dioxide asphyxiation. Licence number LA1230281.

*Received 5 July 1999*
*Revised 17 September 1999*
*Accepted 22 September 1999*

*Correspondence to:* K Vanderkerken

### 5TMM lines and preparation of the 5T2MM cells

The 5TMM lines originated spontaneously in ageing C57BL/KaLwRij mice as described previously (Radl et al, 1979). For the preparation of the cells, BM was flushed from the hind legs. Cells were purified by gradient centrifugation on Lympholyte M (Cedarlane, Hornby, Ontario, Canada) at 450 ***g*** for 25 min, washed and further purified by a Percoll (Pharmacia AB, Uppsala, Sweden) 60% gradient centrifugation at 500 ***g*** for 25 min. Purity was assessed by FACS-staining with 5T2MM anti-idiotype-specific antibodies as described previously (Vanderkerken et al, 1997).

### Injection of the chromium-51 labelled 5T2MM cells

5T2MM cells were prepared as described above and incubated for 80 min at 37°C with 150 μCi chromium-51 (Amersham, Gent, Belgium) for $6 \times 10^6$ cells. After washing, cell viability was assessed by trypan blue exclusion and $2 \times 10^6$ viable cells were injected intravenously (i.v.) (tail vein). After 1, 2 and 18 h mice were sacrificed, blood and urine collected, and the legs, ribs, vertebrae, intestines, kidneys, testes, liver, lungs and heart removed. Following rinsing abundantly in phosphate-buffered saline (PBS) and weighing, the radioactivity of the different organs was assessed in a γ-counter (MR480C/TP ITM, Van Hopplynus, Brussels, Belgium). Results are presented as the percentage of total recovered radioactivity.

### Selective elimination of Kupffer cells in vivo

Kupffer cells (KC) were eliminated in vivo by the ‘liposome mediated macrophage suicide’ technique (Van Rooijen, 1989). With this technique, liposomes are phagocytosed by the macrophages, followed by disruption of the phospholipid bilayers of the liposomes under the influence of lysosomal phospholipases. As a result, clodronate accumulates intracellularly and causes irreversible damage to the macrophages followed by killing of these cells. Non-phagocytic cells are not affected by this method. Then, 0.1 ml of liposomes encapsulating the toxic compound dichloromethylene-diphosphonate ($Cl_2MDP$) were injected i.p. 24 h before the experiment. The liposomes were kindly donated by Dr N Van Rooijen (Free University Amsterdam, The Netherlands).

### Quantitation of KCs in sections

Newly sacrificed mice were subjected to a preperfusion with PBS through the portal vein, followed by perfusion fixation with 1.5% glutaraldehyde in 0.14 M cacaodylate during 4 min at a rate of 4 ml $min^{-1}$. Blocks of liver were cut with a vibratome and rinsed in PBS at 4°C, followed by incubation in peroxidase-medium (0.1 % diaminobenzidine-hydrochloride and 0.02% hydrogen peroxide in 0.1 M PBS, pH 6.9) for 60 min. This was followed by osmium fixation and epon-embedding before ultrathin sectioning. The presence of endogenous peroxidase-positive KC was analyzed by transmission electron microscopy (Philips EM 400, Eindhoven, The Netherlands).

### Isolation and culture of liver non-parenchymal and endothelial cells

Liver endothelial cells (LEC) and non-parenchymal cells (LEC plus KCs) were isolated essentially as described by Smedsrod and Pertoft (1985). Briefly, the portal vein was perfused with Ca-free buffer (‘perfusion buffer’) followed by 0.05% collagenase (Boehringer Mannheim, Scandinavia) in 1% bovine serum albumin (BSA). The resulting cell suspension was washed at 53 ***g*** (2 min) to remove parenchymal cells. The supernatant was centrifuged at 1316 ***g*** (10 min) and cells resuspended in perfusion buffer containing 1% BSA followed by centrifugation through a discontinuous Percoll gradient consisting of 20 ml 50% Percoll and 18 ml 25% Percoll (1316 ***g*** for 30 min). Cells banding at the 25–50% Percoll interface were washed once with perfusion buffer and the pellet was resuspended in RPMI-1640. Non-parenchymal cells were cultured on 24-well tissue plates (Falcon) precoated with human serum fibronectin (0.3 mg $ml^{-1}$ in PBS, 2 mM EDTA, 0.02% azide, a gift from Berit Hansen, University of Tromso), at a density of $1.5 \times 10^6$ cells $well^{-1}$ in 0.5 ml. After 45-min incubation, monolayers were washed three times and incubation was continued in 0.5 ml RPMI-1640 for 2–3 h.

To obtain purified cultures from LEC, non-parenchymal cells were seeded in NUNC dishes (56.7 $cm^2$, NUNC, Heigar, Oslo, Norway). Following incubation at 37°C for 20 min, non-adherent cells (LEC) were collected and seeded in 24-well tissue culture plates precoated with fibronectin. After 45-min incubation, the adherent cells were washed three times with PBS and incubated with 0.5 ml RPMI-1640 for 2–3 h before use.

### Bone marrow and lung endothelial cell lines

STR-4, STR-10, STR-12, murine bone marrow EC lines and LEISVO, a lung EC line were used. All lines were previously established by transfecting primary EC cultures with SV40 (Imai et al, 1999) and were maintained in culture in RPMI-1640 (Gibco, Life Technologies, Gent, Belgium) supplemented with penicillin–streptomycin, glutamine and MEM (Gibco) and 10% bovine serum (Fetal Clone I, Hyclone, UT, USA).

### Adhesion assay

5T2 MM cells (with an average purity of 90%) were labelled with $^{51}Cr$, as described above. Then, $3 \times 10^5$ $^{51}Cr$ labelled tumoral cells were added (0.5 ml of cell suspension per well) to monolayers of freshly isolated LEC or non-parenchymal cells and to BM EC (STR-4, STR-10, STR-12) and lung EC (LEISVO) lines and were incubated for 1 h at 37°C. Supernatant was removed and the cells washed carefully with 0.5 ml of warm PBS. Cell lysates of the adherent cells were obtained by adding 1% sodium dodecyl sulphate (SDS) solution. Radioactivity was assessed in a γ-counter. The percentage adhesion was calculated as follows:

$$\frac{\text{test release}}{\text{total release} - \text{spontaneous release}} \times 100$$

### Liver digestion

After sacrifice, the liver was preperfused through the vena porta with Gey’s balanced salt solution (GBSS) at 37°C for 5 min. This was followed by a perfusion of GBSS supplemented with $7.8 \times 10^{-4}$ M $Ca^{2+}$ containing 0.012% collagenase-P (Boehringer, Brussels, Belgium), 0.20% pronase E (Merck, Overijse, Belgium) and 0.01% DNAase-I (Boehringer) for 30 min at 37°C. During this digestion the liver was kept warm with infra-red light. After perfusion, the liver was removed, dispersed and homogenized in a

© 2000 Cancer Research Campaign mixture of GBSS with $Ca^{2+}$, 0.005% collagenase-P, 0.025% pronase E and 0.001% DNAase-I. Digestion was continued by shaking at 37°C. After centrifugation, cell suspension was filtered through a nylon gauze with 106 mesh.

### Immunogold silver staining on cytospins

Immunogold silver staining was performed with the specific anti-5T2MM idiotype antibodies as described previously (Vanderkerken et al, 1997). Briefly, cytospins of the cell suspensions were prepared at a density of $4 \times 10^5$ cells $ml^{-1}$. The dried cytospins were incubated with a mouse anti-5T2 idiotype antibody ($IgG_1$, 3 μg $ml^{-1}$) for 30 min, followed by rinsing and an incubation with gold-labelled goat-anti mouse $IgG_1$ antibody (Auroprobe LM, Amersham International, UK) at a dilution of 1:75 for another 30 min. After rinsing, a silver enhancement was performed with the Intense B1 Silver Enhancement kit (British Biocall International, Sanvertech, Cardiff, UK) for 15 min at 26°C followed by counterstaining with May–Grünwald–Giemsa. Irrelevant isotype-matched antibodies were used as a control for the first step.

### FACS analysis

Purity of 5T2MM cells was measured by indirect FACS staining. Hereby, $2.5 \times 10^5$ cells were incubated with biotinylated anti-5T2MM (Vanderkerken et al, 1997) or isotype matched control antibodies for 30 min at 4°C. Streptavidin–phycoerythrin (Pharmingen, San Diego, CA, USA) was used as a second step. After each incubation cells were washed twice with PBS supplemented with 1% BSA and 0.02% sodium azide. Cells were fixed in 2% paraformaldehyde after the final washing step and analyzed with a FACSort flowcytometer (Beckton Dickinson, Mountain View, CA, USA). Data of 10 000 events were acquired and stored in list moded files. FACS analysis was performed with LYSIS II software (Beckton Dickinson).

### Nucleic acid preparations

Using TRIzol reagent (Gibco, Life Technologies) total RNA and DNA were extracted from crushed tissue from the BM, lungs, liver, heart, spleen, intestines, kidneys and testis. Two micrograms of total RNA were converted into firststrand cDNA using the Superscript Preamplification System (Gibco, Life Technologies). For each PCR firststrand cDNA corresponding to 50 ng RNA was used.

### PCR analysis

For the detection of the 5T2 $V_H$ sequences (Zhu et al, 1998) a PCR was performed making use of an antisense FR4 primer (5′-GGGGGATCCTGCAGAGACAGTGACCAGAGT) in combination with a sense FR1 primer (5′-GGGGGATCCACAGATCCAG-TTGGTGCAGT) in a 25 μl reaction containing: 2 mM magnesium chloride, 0.2 mM dNTP, 0.6 μM of each primer and 1.25 U *Taq* polymerase (Gibco, Life Technologies). Forty PCR cycles were applied after a 5 min incubation at 94°C with the following steps: 30 s at 94°C, 30 s at 55°C and 30 s at 72°C.

To increase the sensitivity of detection, 10 μl of the PCR mixture was subjected to electrophoresis through a 1.5% agarose/TBE gel, transferred and cross-linked to a nylon membrane (Fluka, Sigma). The filters were hybridized to an internal $^{32}P$-end labelled CDR3 oligonucleotide in 6 × SSC (saline-sodium citrate), 1% SDS, 2.5 × Denhardt's solution and 0.5 × Ppi for 4 h at 42°C. Post-hybridization washes were performed in 2 × SSC/0.1% SDS at 42°C.

In order to assess the sensitivity of our PCR strategy to detect 5T2MM cells in different invaded organs (bone marrow, liver and spleen), tumour cells were serially diluted in bone marrow, spleen cells and hepatocytes (isolation as described above) with a factor of 3.16 and pelleted. Uzing Trizol reagent the RNA was extracted from the cell pellets and a one-step RT-PCR was performed using the Titan™ One Tube RT-PCR System (Boehringer Mannheim, Roche, Switzerland) with the same primers as described above. The analysis of the amplification products and the hybridization with the 5T2MM-specific oligonucleotides were performed as described.

### Statistical analysis

Results are given as the mean and standard deviation. The unpaired Student's *t*-test was used. *P*-values < 0.05 were considered as statistically significant.

### RESULTS

### Injection of radiolabelled cells

The immediate homing of the $^{51}Cr$-labelled 5T2MM cells was assessed by monitoring the radioactivity of the different organs (Figure 1). One hour after injection radioactivity was observed in liver, lungs, BM and kidney. During the experiment (2–18 h) the radioactivity increased in BM, liver and spleen and decreased in the lungs. The radioactivity of 50 μl heparanized blood was extrapolated to 2 ml blood and contained an average of 4.9% of total radioactivity recovered. Radioactivity observed in the kidneys throughout the experiment could be explained by secretion of spontaneously released $^{51}Cr$ in the urine (significant amounts present, data not illustrated).

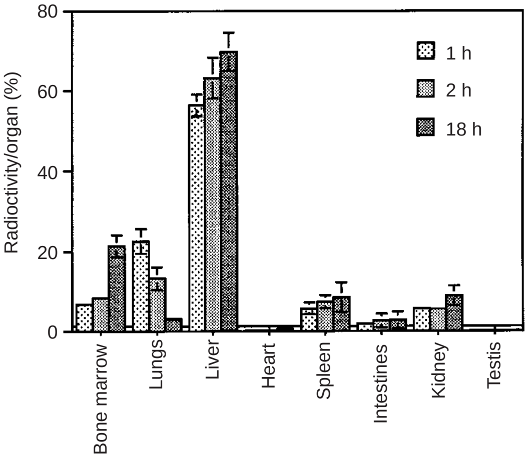

**Figure 1** Radioactivity of different organs 1, 2 and 18 h after intravenous injection of $^{51}Cr$-labelled 5T2MM cells. Each bar represents the mean and standard deviation of four mice. BM represents the radioactivity of ribs, vertebrae and fore and hind legs

*© 2000 Cancer Research Campaign* 

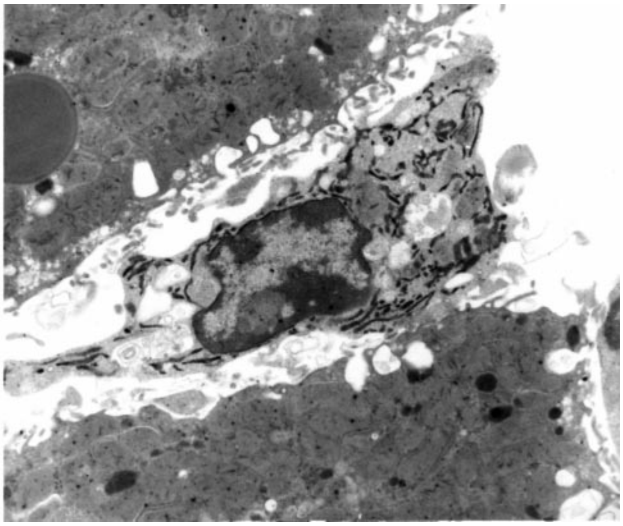

Figure 2 Electron micrograph illustrating a Kupffer cell stained for endogenous peroxidase. Bar = 1 µm

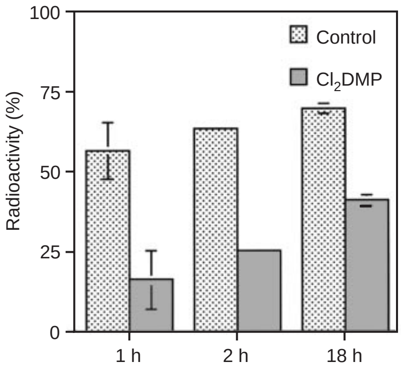

Figure 3 Decrease in radioactivity after pretreatment of the mice with $Cl_2DMP$. Each bar represents the mean and standard deviation of four mice

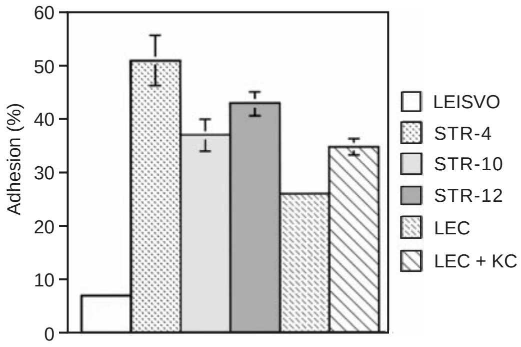

Figure 4 Adhesion of 5TMM cells to bone marrow endothelial cell lines (STR4, STR10, STR12), lung endothelial cells and freshly isolated liver endothelial cells, with (EC + KC) and without the presence of Kupffer cells (EC). Results represent mean and standard deviation of three independent experiments. The difference between LEC + KC and LEC is statistically significant ($P < 0.002$)

### Elimination of KCs

Radioactivity observed in the liver (Figure 1) could be due to phagocytosis of $^{51}$Cr-labelled 5T2MM cells by KCs, the liver resident macrophages. To investigate this possibility KCs were eliminated prior to injection of the labelled 5T2MM cells by injection of liposomes encapsulating the toxic compound $Cl_2DMP$. As a control for the elimination of the KCs, mice were sacrificed and KCs were stained by endogenous peroxidase staining (Figure 2). A total disappearance of the KCs was observed in the treated mice when compared to control mice (data not shown). When $^{51}$Cr-labelled 5T2MM labelled cells were injected in $Cl_2DMP$-treated mice liver radioactivity decreased by 70, 60 and 42% respectively 1, 2 and 18 h after injection (Figure 3). All differences were statistically significant ($P < 0.05$).

### Adhesion assay

Adhesion assays of 5T2MM cells to monolayers of bone marrow-derived EC lines (STR4, STR10 and STR12) and freshly isolated liver EC revealed an adhesion between 30 and 50% while almost no adhesion (7%) was observed to lung-derived EC. The adhesion to liver EC was increased by more than 10% when KCs and EC together made up the monolayer (Figure 4).

### Immunostaining of cells isolated from liver, BM and spleen

To confirm the results obtained with radioactive cells, mononuclear cells were prepared from BM and spleen 18 h after the injection of the non-radioactive 5T2MM cells. Liver was digested enzymatically (collagenase/pronase) and cells were enriched by Lympholyte M and Percoll 60% gradient centrifugation. For BM and spleen respectively 0.8% ± 0.3 (mean ± standard deviation of three independent experiments) and 1.4% ± 0.4 of 5T2 idiotype-positive MM cells were observed by FACS analysis. Since the liver suspension contains a high proportion of autofluorescent cells which interfere with the detection of the small proportion of 5T2MM cells present, cytospins were prepared of the enriched cell suspension and immunogold-silver staining was performed. Figure 5 illustrates a 5T2MM-positive cell present in liver suspension. A mean of 3.2% ± 1.4 positive cells were detected, with all irrelevant control antibodies being negative.

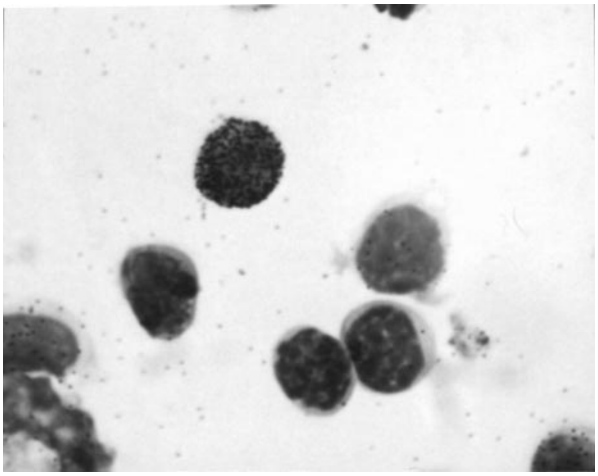

Figure 5 Micrograph illustrating a 5T2MM idiotype-positive cell in a liver suspension, 18 h after injection. Bar = 10 µm

 © 2000 Cancer Research Campaign

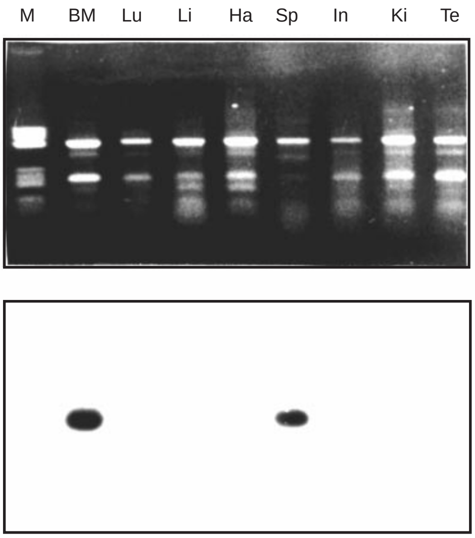

**Figure 6** Amplification with actin primers (**A**) and with 5T2MM-specific primers followed by hybridizations of the PCR-products with the CDR3 probe (**B**) The analysis was performed on first strand cDNA extracted from the following organs: BM (BM), lungs (Lu), liver (Li), heart (Ha), spleen (Sp), intestines (In), kidney (Ki), testis (Te)

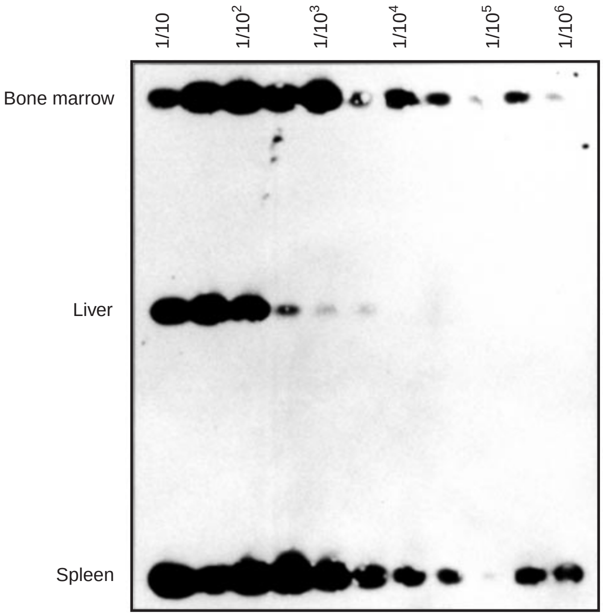

**Figure 7** One step RT-PCR with 5T2MM-specific primers on RNA from 5T2MM cells serially diluted in normal bone marrow, liver and spleen cells and subsequent hybridization of the amplification products with the CDR3 probe

### Detection of 5T2 sequences in different organs using a PCR-based strategy

Using a sensitive PCR strategy, BM, lungs, liver, heart, spleen, intestines, kidney and testes were screened for the presence of tumour cells 18 h after i.v. injection. After extraction of RNA from the different (intact) organs a RT-PCR was performed to amplify part of the tumoural $V_H$ sequence. To increase the sensitivity as well as the specificity of detection, the amplification products obtained were hybridized with the 5T2MM-specific internal CDR3 probe.

Figure 6 illustrates the results of this screening. While the control PCR (Figure 6A) shows an adequate quality of the first strand cDNAs used, the presence of tumoural sequences was shown to be restricted to the samples derived from the BM and the spleen (Figure 6B). Since in the previous experiments the presence of 5T2MM cells had been demonstrated in BM, spleen and liver, the 5T2MM-specific PCR was performed on cDNA (RNA) from 5T2MM cells serially diluted in cells prepared from the organs mentioned above. For BM and spleen we were still able to obtain tumour-specific amplification products in a dilution of 1 tumour cell in $10^6$ normal organ-derived cells. In the liver dilution, however, no 5T2 specific sequences could be demonstrated in the samples containing dilutions beyond 1 tumour cell per 3160 hepatocytes (Figure 7).

### DISCUSSION

MM represents a B-cell malignancy characterized by the presence of a monoclonal population of end-stage B-cells in the BM. Although fully matured BM plasma cells are the predominant cell type in MM, there is much evidence that more immature B-cells are also included in the malignant clone (Bakkus et al, 1992; Van Riet, 1989). The fact that these cells, which are considered to be myeloma precursor cells, are detectable in the blood circulation and that their numbers increase with disease progression makes it very likely that they represent the component that mediates disease progression (Pope et al, 1997).

Although the organ-specific occurrence of cancers and their metastases have been recognized for more than a century, the exact mechanisms have not yet been elucidated. One of the earliest hypotheses was the ‘seed and soil’ hypothesis whereby particular tumour cells (‘seeds’) find organs with a suitable microenvironment (‘soil’) for survival and growth. Since plasmablasts or plasmacytic cells migrate from the blood to the extravascular compartment of the BM it can be assumed that MM cells, which are derived from normal B-cells, have an important part of their migration and homing programme in common with their normal counterparts. The restricted localization of MM cells in the BM, during the initial stage of the disease, could be explained by such a selective migration of the circulating MM cells to the BM and/or by the presence of a unique local microenvironment supporting the survival and growth of the tumoural cells which is not present at extramedullary sites. It is well established that the growth regulation of myeloma cells in the BM compartment is regulated by a functional interplay between the tumour cells and the surrounding stroma involving the action of several cytokines, adhesion molecules and metalloproteinases (Van Riet et al, 1998, review). However, very little is known about the migration behaviour of the MM cells. The purpose of this work was to determine whether the selective localization of MM cells in the BM is due to such a selective homing process, or whether the homing to tissues is more random.

In the first series of experiments the initial distribution of the 5T2MM cells was determined after i.v. injection of radioactively labelled cells. Shortly (1 h) after injection, lungs, liver, BM and spleen were positive. Radioactivity of the lungs decreased in time and was almost completely lost by 18 h. The initial elevated radioactivity of the lungs can be explained by a mechanical

*© 2000 Cancer Research Campaign* 

trapping of the MM cells in the lungs, since after intravenous injection the lungs represent the first vascular bed encountered. Our adhesion assay confirmed that there was only little stable adhesion to lung EC. During the entire experiment (1, 2 and 18 h) the majority of the radioactivity was found in the liver. When a similar experiment was performed for 5T33MM cells, an analogous organ distribution pattern was observed (manuscript in preparation). High liver radioactivity was not due to uptake of released radio-isotope since it is known that sodium chromate ($Na_2{}^{51}CrO_4$) is taken up by living cells in the hexavalent form and is released from lysed cells in the trivalent form, which is not reutilized (Coligan et al, 1984). Another possible explanation for the high radioactivity in the liver was the phagocytosis of labelled 5T2MM cells by KCs, the hepatic resident macrophages. To examine this possibility, KCs were depleted selectively in vivo by the administration of liposome encapsulated clodronate (Van Rooijen, 1989, 1996) prior to injection of the labelled 5T2MM cells, resulting in a decreased accumulation of radioactivity in the liver. This could be partially explained by the adhesion assays which demonstrate an enhanced adhesion of 5T2MM cells to liver endothelium in the presence of KCs and partially by phagocytosis of death labelled tumoural cells.

As a confirmation of the first series of experiments BM, spleen and liver cells were isolated 18 h after injection of the 5T2MM cells and analyzed for the presence of 5T2MM idiotype-positive cells. FACS analysis showed 5T2MM-positive cells in BM and spleen samples, while gold-silver staining on cytospins demonstrated 5T2MM-positive cells in the hepatic cells. As a second confirmation for the selective presence of the MM cells, different organs were removed 18 h after injection of the tumour cells, RNA was prepared and a PCR was performed amplifying specific 5T2MM sequences. These data showed a 5T2MM-specific amplification product in BM and spleen and not in liver. Since the negativity of the liver was clearly in contrast to the previous experiments, the sensitivity level to detect 5T2 sequences via PCR was compared. Therefore 5T2MM cells were serially diluted in BM, spleen cells and hepatocytes with a factor of 3.16. Applying the PCR strategy described above with a subsequent hybridization of the amplification products, we show a more limited 5T2MM-detection in the liver with a factor of at least 300 when compared to BM and spleen. A likely explanation for this limited sensitivity might be the high RNA content of hepatocytes (De Bleser et al, 1997) when compared to other cells. Another explanation for the discrepancy between the different experiments is the size of the organ. In the liver, the 5T2MM cells are diluted in a higher number of cells when compared to BM and spleen. Moreover, it is likely that the MM cells in the liver are not viable since no growth of MM cells was observed in diseased mice (Vanderkerken et al, 1997). Therefore, it is plausible that the MM cells which were trapped mechanically in the liver sinusoids adhere to the hepatic sinusoidal EC but do not survive. The adherence to hepatic EC could be mimicked by in vitro adhesion assays. These results are in agreement with other reports: both activated and memory cells (Smith and Ford, 1983; Jaeschke and Smith, 1997; Tietz and Hamann, 1997; Salmi et al, 1998) and lymphoma cells (Aoudjit et al, 1998; Jonas et al, 1998) display a high affinity for the liver. Moreover IGF-1, which is abundantly secreted in liver and BM, is a chemotactic factor for 5T2MM cells (Vanderkerken et al, 1999) and 5T33MM cells (manuscript in preparation). Although both 5T2 and 5T33MM cells have a similar initial distribution pattern, these cells have a different organ distribution in diseased mice (Vanderkerken et al, 1997). While the 5T2MM cells were only found in BM and part of the spleens of the diseased mice, 5T33MM cells also grow in the liver. One hypothesis for this differential growth in the liver could be a post-homing event, being the differential responsiveness of the 5T2MM cells to the mitogenic stimulus of IGF-1 (no proliferation observed, Vanderkerken et al, 1999) in contrast to 5T33MM cells (proliferation observed, manuscript in preparation). Around mid-gestation, haematopoietic progenitors are thought to migrate in the bloodstream to colonize the liver. At birth the BM becomes the main site for haematopoiesis and remains so throughout adult life. A hierarchy of homing sites may therefore exist in which BM has a higher affinity than either fetal liver or spleen (Blair and Thomas, 1997). Under certain circumstances, however, the liver might take over the role of haematopoiesis (Tanigushi et al, 1996). It has furthermore (Cardier and Barbera-Guillem, 1997) been demonstrated that specific liver sinusoidal EC can support haematopoiesis, in analogy to BM culture.

The unique pathway that underlies the specific presence of MM cells in this model is thus governed by a combination of multiple factors. It is apparent that the first critical step is determined by the presence or up-regulation of adhesion receptors on the MM cells and their specific ligands on the luminal aspect of the EC lining the BM vessels. Our in vitro adhesion assays presented here clearly demonstrate the restricted adhesion to BM EC, similar to the results obtained with progenitor cells (Imai et al, 1999). Several reports describe the specific homing of progenitor cells to the BM compartment. It has become clear that the migration of these progenitors to the BM is restricted by adhesion mechanisms. For both human and mouse lymphocytes and haematopoietic progenitors, VCAM-1, which is expressed on the bone marrow vasculature, serves as one of the main ligands for $\alpha_4$ integrins in the BM (Frenette et al, 1998; Mazo et al, 1998; Berlin-Rufenach et al, 1999; Imai et al, 1999). It is likely that MM cells, which express VLA-4 ($\alpha_4\beta_1$) bind to VCAM-1 expressed on the BM EC during adhesion to and passage through the endothelial barrier. Both in vitro and in vivo experiments will be performed with the 5TMM model in order to elucidate the role of VCAM-1 in the homing of MM cells. Moreover, the local microenvironment is also assumed to be involved in the up-regulation or the induction of conformational changes in, for example, the integrin expression. One class of molecules responsible are the chemokines which bind to transmembranic G-protein-linked receptors triggering conformational changes in leucocyte integrins resulting in a firm, stationary adhesion, followed by leucocyte transmigration through inter-endothelial junctions (Bianchi et al, 1997) or in some cases by trans-endothelial migration (Feng et al, 1998). Once in the extravascular compartment anti-apoptotic and growth stimulating signals should be provided by the stromal microenvironment. In vitro studies demonstrated the inhibition of apoptosis of a human MM-derived cell line in the presence of BM stromal cells (Van Riet et al, 1997). Interleukin 6 has furthermore been described as one of the major survival and growth factors of MM in the BM microenvironment (Kawano et al, 1988; Klein et al, 1989; Uchiyama et al, 1993; Hardin et al, 1994; Juge-Morineau et al, 1995).

We conclude from the present study that the selective presence of 5T2MM cells in the BM and spleen is due to a combination of selective adhesion to vascular endothelium (followed by extravasation to

© 2000 Cancer Research Campaign the extravascular compartment), and of anti-apoptotic and mitogenic signals. The local microenvironment in the BM probably has a unique anti-apoptotic and growth stimulatory feature for the 5T2MM cells when compared to the liver. In the near future we will try to identify these unique properties of the BM.

### ACKNOWLEDGEMENTS

We thank Wendy Van de Voorde and Marijke Baekeland (Dept. CYTO) for their excellent technical assistance. Dr. Yves St-Pierre (Institut Armand Frappier, Laval, QC, Canada) and Dr. Radl (Leiden, The Netherlands) are thanked for helpful discussions. The work was financially supported by the Onderzoeksraad-Vrije Universiteit Brussel (OZR-VUB), Fonds voor Wetenschappelijk Onderzoek Vlaanderen (FWO-Vl), Vlaamse Liga tegen Kanker and The International Myeloma Foundation (Brain Novis Award 1998). Karin Vanderkerken is a postdoctoral fellow and Isabelle Vande Broek is a research assistant of FWO-Vl.

### REFERENCES

Aoudjit F, Potworowski EF and St-Pierre Y (1998) The metastatic characteristics of murine lymphoma cell lines in vivo are manifested after target organ invasion. *Blood* **91**: 623–629

Bakkus MH, Heirman C, Van Riet I, Van Camp B and Thielemans K (1992) Evidence that multiple myeloma Ig heavy chain VDJ genes contain somatic mutations but show no intraclonal variation. *Blood* **80**: 2326–2335

Berlin-Rufenach C, Otto F, Mathies M, Westermann J, Owen MJ, Hamann A and Hogg N (1999) Lymphocyte migration in lymphocyte function-associated antigen (LFA-1)-deficient mice. *J Exp Med* **9**: 1467–1478

Bianchi E, Bender JR, Blasi F and Pardi R (1997) Through and beyond the wall: late steps in leukocyte transendothelial migration. *Immunol Today* **18**: 586–591

Blair A and Thomas DB (1997) Preferential adhesion of fetal liver derived primitive haemopoietic progenitor cells to bone marrow stroma. *Br J Haematol* **99**: 726–731

Butcher EC and Picker LJ (1996) Lymphocyte homing and homeostasis. *Science* **272**: 60–66

Cardier JE and Barbera-Guillem E (1997) Extramedullary hematopoiesis in adult liver is associated with specific sinusoidal endothelial cells. *Hepatology* **26**: 165–175

Coligan JE, Kruisbeek AM, Margulies DH, Shevach EM and Strober W (1984) Induction and measurement of cytotoxic T lymphocyte activity. In: *Current Protocols in Immunology*, Coico R (ed) pp. 3–11.14 Wiley Interscience

De Bleser P, Niki T, Rogiers V and Geerts A (1997) Transforming growth factor-β gene expression in normal and fibrotic rat liver. *J Hepatol* **26**: 886–893

Feng D, Nagy JA, Pyne K, Dvorak HF and Dvorak AM (1998) Neutrophils emigrate from venules by a transendothelial cell pathway in response to FMLP. *J Exp Med* **187**: 903–915

Frenette PS, Subbarao S, Mazo IB, von Adrian UH and Wagner DD (1998) Endothelial selectins and vascular cell adhesion molecule-1 promote hematopietic progenitor homing to bone marrow. *Proc Natl Acad Sci USA* **95**: 14423–14428

Hallek M, Bergsagel PL and Anderson KC (1998) Multiple myeloma: increasing evidence for a multistep transformation process. *Blood* **91**: 3–31

Hardin J, MacLeod S, Grigorieva I, Chang R, Barlogie B, Xiao H and Epstein J (1994) Interleukin-6 prevents dexamathasone-induced myeloma cell death. *Blood* **84**: 3063–3070

Imai K and Kobayashi M (1998) Differences between bone marrow and lung endothelial cells. *Semin Thromb Hemostas* **24**: 275–277

Imai K, Kobayashi M, Wang J, Ohiro Y, Hamada JI, Cho Y, Imamura M, Musashi M, Kondo T, Hosokawa M and Asaka M (1999) Selective transendothelial migration of hematopoietic progenitor cells: a role in homing of progenitor cells. *Blood* **93**: 149–156

Jaeschke H and Smith CW (1997) Cell adhesion and migration. III. Leukocyte adhesion and transmigration in the liver vasculature. *Am J Physiol* **273** (*Gastrointest. Liver Physiol* 36): G1169–1173

Jonas P, Holzmann B, Jablonski-Westrich D and Hamann A (1998) Dissemination capacity of murine lymphoma cells is not dependent on efficient homing. *Int J Cancer* **77**: 402–407

Juge-Morineau N, Francois S, Puthier D, Godard A, Bataille R and Amiot M (1995) The gp130 family cytokines IL-6, LIF and OSM but not IL-11 can reverse the anti-proliferative effect of dexamethasone on human myeloma cells. *Br J Haematol* **90**: 707–710

Kawano M, Hirano T, Matsuda T, Taga T, Horii Y, Iwato K, Asaoku H, Tang B, Tanabe O, Tanaka H, Kuramoto A and Kishimoto T (1988) Autocrine generation and requirement of BSF-2/I1-6 for human multiple myelomas. *Nature* **332**: 83–85

Klein B, Zhang XG, Jourdan M, Content J, Houssiu F, Aarden L, Piechaczyk M and Bataille R (1989) Paracrine rather than autocrine regulation of myeloma-cell growth and differentation by interleukin-6. *Blood* **73**: 517–526

Mazo IB, Gutierrez-Ramos JC, Frenette PS, Hynes RO, Wagner DD and von Andrian UH (1998) Hematopoietic progenitor cell rolling in BM microvessels: parallel contributions by endothelial selectins and vascular adhesion molecule 1. *J Exp Med* **188**: 465–474

Pope B, Brown R, Gibson J and Joshua D (1997) Plasma cells in peripheral blood stem cell harvest from patients with multiple myeloma are predominantly polyclonal. *Bone Marrow Transplant* **20**: 205–210

Radl J, De Glopper E, Schuit HRE and Zurcher C (1979) Idiopathic paraproteinemia. II. Transplantation of the paraprotein-producing clone from old to young C57BL/KaLwRij mice. *J Immunol* **122**: 609–613

Salmi M, Adams D and Jalkanen S (1998) Cell adhesion and migration. IV. Lymphocyte trafficking in the intestine and liver. *Am J Physiol* **274** (*Gastrointest. Liver Physiol*. 37): G1–G6

Smedsrod B and Pertoft H (1985) Preparation of pure hepatocytes and reticuloendothelial cells in high yield from a single rat liver by means of Percoll centrifugation and selective adherence. *J Leukoc Biol* **38**: 213–230

Smith ME and Ford WL (1983) The recirculating lymphocyte pool of the rat: a systematic description of the migratory behaviour of recirculating lymphocytes. *Immunology* **49**: 83–94

Tanigushi H, Toyoshima T, Fukao K and Nakauchi H (1996) Presence of hematopoietic stem cells in the adult liver. *Nat Med* **2**: 198–203

Tietz W and Hamann A (1997) The migratory behaviour of murine CD4+ cells of memory phenotype. *Eur J Immunol* **27**: 2225–2232

Uchiyama H, Barut KA, Mohrabacher AF, Chauchan D and Anderson KC (1993) Adhesion of human myeloma-derived cell lines to bone marrow stromal cells stimulates interleukin-6 seceretion. *Blood* **82**: 3712–3720

Van Riet I, Heirman C, Lacor P, De Waele M, Thielemans K and Van Camp B (1989) Detection of monoclonal B lymphocytes in bone marow and peripheral blood of multiple myeloma patients by immunoglobulin gene rearrangements studies. *Br J Haematol* **73**: 289–295

Van Riet I, De Greef C, Aharchi F, Woischwill C, De Waele M, Bakkus M, Lacor P, Schots R and Van Camp B (1997) Establishment and characterization of a human stroma-dependent myeloma cell line (MM5.1) and its stroma-independent variant (MM5.2). *Leukemia* **11**: 284–293

Van Riet I, Vanderkerken K, De Greef C and Van Camp (1998) Homing behaviour of the malignant cell clone in multiple myeloma. *Medical Oncology* **15**: 154–164

Vanderkerken K, Goes E, De Raeve H, Radl J and Van Camp B (1996) Follow-up of bone lesions in an experimental multiple myeloma mouse model: description of an in vivo technique using radiography dedicated for mammography. *Br J Cancer* **73**: 1463–1465

Vanderkerken K, De Raeve H, Goes E, Van Meirvenne S, Radl J, Van Riet I, Thielemans K and Van Camp B (1997) Organ involvement and phenotypic adhesion profile of 5T2 and 5T33 myeloma cells in the C57BL/KaLwRij mouse. *Br J Cancer* **76**: 451–460

Vanderkerken K, Asosingh K, Braet F, Van Riet I and Van Camp B (1999) Insulin like growth factor-1 acts as a chemoattractant factor for 5T2 multiple myeloma cells. *Blood* **93**: 235–241

Van Rooijen N (1989) The liposome-mediated 'suicide' technique. *J Immunol Methods* **124**: 1–6

Van Rooijen N and Sanders A (1996) Kupffer cell depletion by liposome-delivered drugs: comparative activity by intracellular clodronate, propamidine, and ethylenediaminetetraacetic acid. *Hepatology* **23**: 1239–1243

Zhu D, Van Arkel C, King CA, Van Meirvenne S, De Greef C, Thielemans K, Radl J and Stevenson K (1998) Immunoglobulin $V_H$ gene sequence analysis of spontaneous murine immunoglobule-secreting B-cell tumors with clinical features of human disease. *Immunology* **93**: 162–170

© 2000 Cancer Research Campaign 
